# Comment on “Neuropsychological Tests of Memory, Visuospatial, and Language Function in Parkinson's Disease: Review, Critique, and Recommendations”

**DOI:** 10.1002/mds.70148

**Published:** 2025-12-16

**Authors:** Joshua P. Woller, Alireza Gharabaghi

**Affiliations:** ^1^ University of Tübingen Tübingen Germany

**Keywords:** Parkinson's disease, neuropsychological assessment, test reliability, sensitivity to change, measurement invariance, cognitive phenotyping

We commend Bezdicek and colleagues for a rigorous, consensus‐based evaluation of neuropsychological tests for memory, language, visuospatial function, and premorbid intelligence in Parkinson's disease (PD).^1^ Their tiered recommendations provide practical guidance for level‐II assessment across Parkinson's Disease Mild Cognitive Impairment (PD‐MCI) and PD dementia (PDD) and for outcome selection in trials.

The panel's core messages are clear. Word‐list and story‐recall measures show acceptable validity, reliability, and sensitivity to change in PD. The Boston Naming Test is a practical reference for language, matrix reasoning is the current visuospatial choice, and National adult reading test (NART) or NAART improves adjudication through premorbid‐ability estimates. These points, including the stated limitations for early‐stage sensitivity and motor confounds, provide a strong foundation for standardized workflows.

We suggest three additions that support the authors' framework.

First, reliability should also be treated as a design constraint of subsequent analyses. The maximum observable association between two variables is limited by their reliabilities. When studies compare or pool correlations across outcomes with unequal reliability, attenuation biases estimate toward zero and can be mistaken for weak construct associations.^2^ Explicit reporting of reliability for each selected measure, and correction for attenuation in secondary analyses where justified, would reduce interpretive variability and improve cross‐cohort comparability.

Second, sensitivity to change should use complementary thresholds. Minimal detectable change quantifies the smallest difference beyond measurement error, and minimal clinically important difference reflects patient‐perceived benefit.^3^ The latter is, therefore, often used when assessing outcomes of clinical trials.^4^ If responder classification is applied, minimally detectable change (MDC) and minimal clinically important difference (MCID) should shape that split. When MCID is smaller than MDC, individual change scores that exceed MCID but fall below MDC remain indeterminate and should not be interpreted as true change. This differentiation is especially relevant given the substantial day‐to‐day variability of PD symptoms, which may surpass MCID values.^5^

Third, measurement invariance should be established before comparing groups, subtypes, therapy states, or time points.^6^ Without item‐level invariance, observed score differences may be driven by a subset of items that function differentially, which decouples scores from the intended construct. Given the paper's critique about uneven domain sensitivity and executive contamination of visuospatial measures, routine checks for differential item functioning and factorial stability would strengthen inferences in longitudinal and multicenter studies.

These additions are consistent with the authors' future directions and further expand the Desiderata for neuropsychological testing in PD patients.^7^ We support the validation of controlled‐learning memory paradigms such as Serial Reaction Time (SRT) or Memory Binding Test (MBT) to minimize executive load. We also support efforts to limit motor confounds in language and visuospatial testing, including digital speech or language analytics for prodromal cohorts. Further, premorbid IQ should be incorporated routinely because it can alter PD‐MCI classification and improve diagnostic precision. Finally, implementation considerations matter. The documented legal and cost barriers argue for standardized, shareable core batteries with validated parallel forms to support equitable uptake across centers.

In sum, this review advances standardization in PD cognitive phenotyping. Embedding reliability‐aware analyses, dual change thresholds, and routine invariance testing will make these recommendations more reproducible and trial‐ready.

## Author Roles

(1) Research Project: A. Conception, B. Organization, C. Execution; (2) Statistical Analysis: A. Design, B. Execution, C. Review and Critique; (3) Manuscript Preparation: A. Writing of the First Draft, B. Review and Critique.

J.P.W.: 1A, 1B, 1C, 3A, 3B.

A.G.: 1A, 1B, 1C, 3A, 3B.

## Data Availability

Data sharing not applicable to this article as no datasets were generated or analysed during the current study.
